# State Medicaid Coverage for Tobacco Cessation Treatments and Barriers to Accessing Treatments — United States, 2008–2018

**DOI:** 10.15585/mmwr.mm6906a2

**Published:** 2020-02-14

**Authors:** Anne DiGiulio, Zach Jump, Stephen Babb, Anna Schecter, Kisha-Ann S. Williams, Debbie Yembra, Brian S. Armour

**Affiliations:** ^1^American Lung Association, Chicago, Illinois; ^2^Office on Smoking and Health, National Center for Chronic Disease Prevention and Health Promotion, CDC.

The prevalence of current cigarette smoking is approximately twice as high among adults enrolled in Medicaid (23.9%) as among privately insured adults (10.5%), placing Medicaid enrollees at increased risk for smoking-related disease and death (*1*). Medicaid spends approximately $39 billion annually on treating smoking-related diseases (*2*). Individual, group, and telephone counseling and seven Food and Drug Administration (FDA)–approved medications* are effective in helping tobacco users quit (*3*). Comprehensive, barrier-free, widely promoted coverage of these treatments increases use of cessation treatments and quit rates and is cost-effective (*3*). To monitor changes in state Medicaid cessation coverage for traditional Medicaid enrollees^†^ over the past decade, the American Lung Association collected data on coverage of nine cessation treatments by state Medicaid programs during December 31, 2008–December 31, 2018: individual counseling, group counseling, and the seven FDA-approved cessation medications^§^; states that cover all nine of these treatments are considered to have comprehensive coverage. The American Lung Association also collected data on seven barriers to accessing covered treatments.^¶^ As of December 31, 2018, 15 states covered all nine cessation treatments for all enrollees, up from six states as of December 31, 2008. Of these 15 states, Kentucky and Missouri were the only ones to have removed all seven barriers to accessing these cessation treatments. State Medicaid programs that cover all evidence-based cessation treatments, remove barriers to accessing these treatments, and promote covered treatments to Medicaid enrollees and health care providers could reduce smoking, smoking-related disease, and smoking-attributable federal and state health care expenditures (*3*–*7*).

During December 31, 2008–December 31, 2018, the American Lung Association compiled data on state Medicaid tobacco cessation coverage from state Medicaid and Medicaid managed care plan member and provider websites and handbooks, policy manuals, plan formularies and preferred drug lists; Medicaid state plan amendments; and relevant regulations and laws.** Analysts searched for mentions of the nine cessation treatments on state Medicaid websites and other relevant state-sponsored websites and the Google search engine. The American Lung Association contacted personnel from state Medicaid agencies, state health departments, or other state government agencies to verify the information collected, retrieve missing documents, and reconcile discrepancies.

As of December 31, 2018, all 50 states and the District of Columbia (DC) covered at least some cessation treatments for all traditional Medicaid enrollees, compared with 46 states and DC as of December 31, 2008. As of December 31, 2018, 16 states covered both individual and group counseling for all enrollees, up from 13 states in December 2008 (Table 1). Thirty-six states^††^ covered all seven FDA-approved cessation medications for all traditional Medicaid enrollees, up from 20 states in December 2008 (Table 2). As of December 31, 2018, 15 states (California, Colorado, Connecticut, Indiana, Kansas, Kentucky, Maine, Massachusetts, Minnesota, Missouri, Ohio, Oregon, Rhode Island, South Carolina, and Wisconsin) covered all nine cessation treatments for all traditional Medicaid enrollees, an increase from six states as of December 31, 2008 (Table 1) (Table 2). Eleven states (California, Colorado, Connecticut, Kansas, Kentucky, Maine, Missouri, Ohio, Rhode Island, South Carolina, and Wisconsin) achieved this comprehensive level of coverage during the study period. Conversely, two states (Nebraska and Pennsylvania) that covered all nine cessation treatments in December 2008 no longer did so in December 2018.^§§^ Thirteen (87%) of the 15 states that covered all nine cessation treatments in 2018 had barriers in place for some treatments (Table 3); the remaining two states (Kentucky and Missouri) have removed all seven barriers examined in this study.

**TABLE 1:** Medicaid coverage for tobacco cessation counseling, by state — United States, 2008 and 2018*^,†^

State	Individual counseling		Group counseling	
	2008	2018	2008	2018
Alabama	P	P	No	No
Alaska	Yes	Yes	No	No
Arizona	No	P	No	No
Arkansas	Yes	Yes	Yes	No
California	Yes	Yes	V	Yes
Colorado	No	Yes	No	Yes
Connecticut	No	Yes	No	Yes
Delaware	No	Yes	No	No
District of Columbia	V	Yes	V	No
Florida	Yes	V	Yes	V
Georgia	No	Yes	No	V
Hawaii	No	Yes	V	V
Idaho	No	Yes	Yes	No
Illinois	No	V	No	No
Indiana	Yes	Yes	Yes	Yes
Iowa	Yes	V	No	V
Kansas	No	Yes	No	Yes
Kentucky	P	Yes	No	Yes
Louisiana	No	Yes	No	V
Maine	Yes	Yes	No	Yes
Maryland	Yes	Yes	Yes	No
Massachusetts	Yes	Yes	Yes	Yes
Michigan	V	Yes	V	V
Minnesota	Yes	Yes	Yes	Yes
Mississippi	P	P	P	V
Missouri	No	Yes	No	Yes
Montana	Yes	Yes	No	No
Nebraska	Yes	Yes	Yes	V
Nevada	Yes	V	Not available	V
New Hampshire	Yes	Yes	P	V
New Jersey	Yes	V	Yes	V
New Mexico	No	V	V	V
New York	P	Yes	P	Yes
North Carolina	No	Yes	No	No
North Dakota	Yes	P	Yes	No
Ohio	No	Yes	No	Yes
Oklahoma	Yes	Yes	No	No
Oregon	Yes	Yes	Yes	Yes
Pennsylvania	Yes	Yes	Yes	V
Rhode Island	Yes	Yes	Yes	Yes
South Carolina	No	Yes	No	Yes
South Dakota	No	P	No	No
Tennessee	No	V	No	No
Texas	V	V	Not available	V
Utah	P	Yes	P	P
Vermont	No	Yes	No	No
Virginia	No	V	P	V
Washington	Yes	V	No	No
West Virginia	No	Yes	V	V
Wisconsin	Yes	Yes	Yes	Yes
Wyoming	Yes	Yes	No	No
**Totals**				
**Yes**	**23**	**36**	**14**	**16**
**No**	**20**	**0**	**24**	**18**
**V**	**3**	**10**	**6**	**16**
**P**	**5**	**5**	**5**	**1**
**Not available**	**0**	**0**	**2**	**0**

**Abbreviations:** No = treatment not covered for any Medicaid enrollee; P = treatment covered for pregnant women only; V = coverage varies, with treatment covered for some, but not all, traditional Medicaid enrollees; Yes = treatment covered for all Medicaid enrollees.

* Data as of December 31, 2008, and December 31, 2018.

^†^ Because of differences in the methods and timing of data collection, some findings differ from findings on this topic published before 2014.

**TABLE 2:** Medicaid coverage for tobacco cessation medications, by state — United States, 2008 and 2018*^,†^

State	NRT patch		NRT gum		NRT lozenge		NRT nasal spray		NRT inhaler		Bupropion (Zyban)		Varenicline (Chantix)	
	2008	2018	2008	2018	2008	2018	2008	2018	2008	2018	2008	2018	2008	2018
Alabama	No	Yes	No	Yes	No	Yes	No	Yes	No	Yes	No	Yes	No	Yes
Alaska	Yes	Yes	Yes	Yes	Yes	Yes	Yes	Yes	No	Yes	Yes	Yes	Yes	Yes
Arizona	Yes	Yes	Yes	Yes	Yes	Yes	Yes	Yes	Yes	Yes	Yes	Yes	Yes	Yes
Arkansas	Yes	Yes	Yes	Yes	No	No	No	No	No	No	Yes	Yes	Yes	Yes
California	Yes	Yes	V	Yes	V	Yes	V	Yes	V	Yes	Yes	Yes	V	Yes
Colorado	Yes	Yes	Yes	Yes	Yes	Yes	Yes	Yes	Yes	Yes	Yes	Yes	Yes	Yes
Connecticut	No	Yes	No	Yes	No	Yes	No	Yes	No	Yes	No	Yes	No	Yes
Delaware	Yes	Yes	Yes	Yes	Yes	Yes	Yes	Yes	Yes	Yes	No	Yes	Yes	Yes
District of Columbia	V	Yes	V	Yes	V	Yes	No	V	No	V	V	Yes	V	V
Florida	Yes	Yes	Yes	Yes	No	Yes	No	No	No	No	Yes	Yes	No	Yes
Georgia	No	Yes	No	Yes	No	Yes	No	V	No	V	No	Yes	No	V
Hawaii	V	Yes	V	Yes	V	V	V	V	V	V	V	Yes	V	Yes
Idaho	Yes	Yes	Yes	Yes	Yes	Yes	Yes	Yes	No	Yes	Yes	Yes	Yes	Yes
Illinois	Yes	Yes	Yes	Yes	Yes	Yes	Yes	V	Yes	V	Yes	Yes	Yes	V
Indiana	Yes	Yes	Yes	Yes	Yes	Yes	Yes	Yes	Yes	Yes	Yes	Yes	Yes	Yes
Iowa	Yes	Yes	Yes	Yes	No	Yes	No	Yes	No	Yes	Yes	Yes	Yes	Yes
Kansas	Yes	Yes	No	Yes	No	Yes	No	Yes	No	Yes	Yes	Yes	Yes	Yes
Kentucky	No	Yes	No	Yes	No	Yes	No	Yes	No	Yes	No	Yes	No	Yes
Louisiana	Yes	Yes	Yes	Yes	No	Yes	Yes	V	Yes	V	Yes	Yes	Yes	V
Maine	Yes	Yes	Yes	Yes	Yes	Yes	Yes	Yes	Yes	Yes	Yes	Yes	Yes	Yes
Maryland	V	Yes	V	Yes	V	Yes	No	Yes	No	Yes	V	Yes	V	Yes
Massachusetts	Yes	Yes	Yes	Yes	Yes	Yes	Yes	Yes	Yes	Yes	Yes	Yes	Yes	Yes
Michigan	Yes	Yes	V	Yes	V	Yes	V	Yes	V	Yes	V	Yes	V	Yes
Minnesota	Yes	Yes	Yes	Yes	Yes	Yes	Yes	Yes	Yes	Yes	Yes	Yes	Yes	Yes
Mississippi	Yes	Yes	Yes	Yes	Yes	Yes	Yes	Yes	Yes	Yes	Yes	Yes	Yes	Yes
Missouri	No	Yes	No	Yes	No	Yes	No	Yes	No	Yes	No	Yes	No	Yes
Montana	Yes	Yes	Yes	Yes	Yes	Yes	Yes	Yes	Yes	Yes	Yes	Yes	Yes	Yes
Nebraska	Yes	Yes	Yes	Yes	Yes	Yes	Yes	Yes	Yes	Yes	Yes	Yes	Yes	Yes
Nevada	Yes	Yes	Yes	Yes	Yes	Yes	Yes	V	Yes	Yes	Yes	Yes	Yes	Yes
New Hampshire	Yes	Yes	Yes	Yes	Yes	Yes	Yes	Yes	Yes	Yes	Yes	Yes	Yes	Yes
New Jersey	Yes	Yes	V	Yes	No	Yes	No	V	No	V	V	Yes	V	Yes
New Mexico	V	Yes	V	Yes	V	Yes	V	V	V	V	V	Yes	V	Yes
New York	Yes	Yes	Yes	Yes	No	Yes	Yes	Yes	Yes	V	Yes	Yes	Yes	Yes
North Carolina	Yes	Yes	Yes	Yes	Yes	Yes	Yes	Yes	Yes	Yes	Yes	Yes	Yes	Yes
North Dakota	Yes	Yes	Yes	Yes	No	Yes	No	Yes	Yes	Yes	Yes	Yes	Yes	Yes
Ohio	Yes	Yes	Yes	Yes	Yes	Yes	Yes	Yes	Yes	Yes	Yes	Yes	Yes	Yes
Oklahoma	Yes	Yes	Yes	Yes	Yes	Yes	Yes	Yes	Yes	Yes	Yes	Yes	Yes	Yes
Oregon	Yes	Yes	Yes	Yes	Yes	Yes	Yes	Yes	Yes	Yes	Yes	Yes	Yes	Yes
Pennsylvania	Yes	Yes	Yes	Yes	Yes	Yes	Yes	V	Yes	V	Yes	Yes	Yes	Yes
Rhode Island	Yes	Yes	Yes	Yes	Yes	Yes	Yes	Yes	Yes	Yes	V	Yes	V	Yes
South Carolina	Yes	Yes	Yes	Yes	Yes	Yes	Yes	Yes	Yes	Yes	Yes	Yes	Yes	Yes
South Dakota	No	No	No	No	No	No	No	No	No	No	Yes	Yes	Yes	Yes
Tennessee	No	Yes	No	Yes	No	Yes	No	Yes	No	Yes	No	Yes	No	Yes
Texas	Yes	Yes	Yes	Yes	No	Yes	Yes	Yes	Yes	Yes	Yes	Yes	Yes	Yes
Utah	V	Yes	V	Yes	V	Yes	V	Yes	V	Yes	Yes	Yes	Yes	Yes
Vermont	Yes	Yes	Yes	Yes	Yes	Yes	Yes	Yes	Yes	Yes	Yes	Yes	Yes	Yes
Virginia	Yes	Yes	Yes	Yes	Yes	V	Yes	V	Yes	V	Yes	Yes	Yes	Yes
Washington	Yes	Yes	Yes	Yes	No	Yes	No	V	No	V	Yes	Yes	Yes	V
West Virginia	V	Yes	V	Yes	V	Yes	V	Yes	V	Yes	No	Yes	No	Yes
Wisconsin	Yes	Yes	Yes	Yes	No	Yes	Yes	Yes	Yes	Yes	Yes	Yes	Yes	Yes
Wyoming	Yes	Yes	Yes	Yes	Yes	Yes	No	Yes	No	Yes	Yes	Yes	Yes	Yes
**Totals**														
**Yes**	**38**	**50**	**34**	**50**	**25**	**47**	**28**	**37**	**27**	**37**	**36**	**51**	**35**	**46**
**No**	**7**	**1**	**8**	**1**	**18**	**2**	**17**	**3**	**18**	**3**	**8**	**0**	**8**	**0**
**V**	**6**	**0**	**9**	**0**	**8**	**2**	**6**	**11**	**6**	**11**	**7**	**0**	**8**	**5**

**Abbreviations:** No = treatment not covered for any Medicaid enrollee; NRT = nicotine replacement therapy; V = coverage varies, with treatment covered for some, but not all, traditional Medicaid enrollees; Yes = treatment covered for all Medicaid enrollees.

* Data as of December 31, 2008, and December 31, 2018.

^†^ Because of differences in the methods and timing of data collection, some findings differ from findings on this topic published before 2014.

**TABLE 3:** Barriers to Medicaid coverage for tobacco cessation treatments, by state — United States, 2008 and 2018*^,†,§^

State	Copayment required		Prior authorization required		Counseling required for medications		Stepped care therapy		Limits on duration		Annual limit on number of quit attempts		Lifetime limit on number of quit attempts	
	2008	2018	2008	2018	2008	2018	2008	2018	2008	2018	2008	2018	2008	2018
Alabama	No	No	Yes	Yes	N/A	No	N/A	No	Yes	Yes	No	Yes	No	No
Alaska	Yes	Yes	Yes	No	Yes	No	Yes	No	Yes	Yes	Yes	Yes	No	No
Arizona	No	No	No	No	No	No	No	No	Yes	Yes	No	Yes	No	No
Arkansas	No	No	Yes	Yes	Yes	Yes	No	No	Yes	V	Yes	Yes	No	No
California	Yes	No	No	V	Yes	No	No	No	Yes	V	Yes	V	No	No
Colorado	Yes	No	Yes	No	Yes	Yes	No	No	Yes	Yes	Yes	Yes	Yes	No
Connecticut	N/A	No	N/A	Yes	N/A	No	N/A	No	N/A	Yes	N/A	No	N/A	No
Delaware	Yes	No	Yes	V	Yes	V	Yes	V	Yes	V	Yes	V	No	No
District of Columbia	No	V	No	V	No	No	No	No	Yes	V	No	V	No	No
Florida	Yes	V	No	No	No	No	Yes	No	V	Yes	V	Yes	V	No
Georgia	N/A	V	N/A	V	N/A	No	N/A	No	N/A	Yes	N/A	Yes	N/A	No
Hawaii	V	No	V	V	V	Yes	V	V	V	V	V	Yes	V	No
Idaho	No	No	No	Yes	Yes	Yes	No	Yes	No	Yes	Yes	Yes	No	No
Illinois	No	V	Yes	V	No	No	No	V	No	V	No	V	No	No
Indiana	Yes	Yes	No	V	Yes	Yes	Yes	V	Yes	Yes	Yes	Yes	No	No
Iowa	Yes	No	Yes	Yes	Yes	Yes	No	Yes	Yes	Yes	Yes	Yes	No	No
Kansas	Yes	No	No	No	No	No	No	No	Yes	Yes	Yes	Yes	No	No
Kentucky	No	No	No	No	N/A	No	N/A	No	Yes	No	No	No	No	No
Louisiana	Yes	V	No	V	Yes	No	No	No	No	V	No	V	No	No
Maine	Yes	No	Yes	Yes	No	No	Yes	Yes	Yes	No	Yes	No	Yes	No
Maryland	V	No	V	Yes	V	No	V	Yes	V	Yes	V	No	V	No
Massachusetts	Yes	Yes	Yes	Yes	No	No	No	No	No	Yes	No	Yes	No	No
Michigan	V	No	V	No	V	No	V	No	V	V	V	No	Not available	No
Minnesota	Yes	No	No	V	No	No	No	No	No	V	No	No	No	No
Mississippi	Yes	V	No	Yes	No	No	No	No	No	No	No	Yes	No	No
Missouri	N/A	No	N/A	No	N/A	No	N/A	No	N/A	No	N/A	No	N/A	No
Montana	Yes	No	Yes	Yes	No	No	Yes	Yes	Yes	Yes	No	Yes	Yes	No
Nebraska	Yes	V	Yes	Yes	Yes	Yes	No	No	Yes	Yes	No	Yes	No	No
Nevada	Yes	No	Yes	V	No	No	No	No	Yes	Yes	Yes	V	No	No
New Hampshire	Yes	V	Yes	V	No	V	No	V	Yes	V	Yes	V	No	No
New Jersey	V	V	V	No	V	No	V	No	V	V	V	V	V	No
New Mexico	No	V	No	V	V	V	No	No	V	V	Yes	Yes	No	No
New York	V	V	No	No	No	No	No	No	Yes	No	Yes	No	No	No
North Carolina	Yes	Yes	No	No	No	No	No	Yes	No	Yes	No	No	No	No
North Dakota	Yes	Yes	Yes	Yes	Yes	Yes	No	Yes	Yes	Yes	Yes	Yes	No	No
Ohio	Yes	No	No	V	No	No	No	V	No	V	No	V	No	No
Oklahoma	Yes	No	Yes	No	Yes	No	No	No	Yes	Yes	Yes	No	No	No
Oregon	Yes	No	No	Yes	No	No	No	No	No	Yes	No	Yes	No	No
Pennsylvania	Yes	V	V	V	No	No	No	V	Yes	V	Yes	V	No	No
Rhode Island	V	No	V	V	Yes	V	No	No	Yes	V	No	No	No	No
South Carolina	Yes	No	Yes	No	No	No	Yes	No	Yes	Yes	Yes	Yes	No	No
South Dakota	Yes	Yes	No	No	No	No	No	No	No	No	No	No	No	No
Tennessee	N/A	Yes	N/A	Yes	N/A	No	N/A	Yes	N/A	Yes	N/A	Yes	N/A	V
Texas	V	No	No	Yes	V	No	V	Yes	Yes	V	Yes	V	No	No
Utah	Yes	Yes	Yes	V	No	V	No	V	Yes	V	No	Yes	Yes	No
Vermont	Yes	No	No	Yes	Yes	No	No	Yes	Yes	Yes	Yes	Yes	No	No
Virginia	Yes	V	No	V	No	No	No	V	No	No	No	No	No	No
Washington	No	No	Yes	V	No	V	No	V	No	V	No	V	No	V
West Virginia	No	No	Yes	Yes	Yes	Yes	No	Yes	Yes	Yes	No	Yes	No	No
Wisconsin	Yes	Yes	No	No	No	No	No	No	No	Yes	No	No	No	No
Wyoming	Yes	Yes	No	No	No	No	No	No	Yes	Yes	Yes	Yes	No	No
**Totals**														
**Yes**	**30**	**10**	**19**	**17**	**15**	**9**	**7**	**11**	**28**	**26**	**21**	**25**	**4**	**0**
**No**	**10**	**28**	**22**	**16**	**24**	**36**	**33**	**30**	**13**	**7**	**21**	**14**	**38**	**49**
**V**	**7**	**13**	**6**	**18**	**6**	**6**	**5**	**10**	**6**	**18**	**5**	**12**	**4**	**2**
**N/A**	**4**	**0**	**4**	**0**	**6**	**0**	**6**	**0**	**4**	**0**	**4**	**0**	**4**	**0**
**Not available**	**0**	**0**	**0**	**0**	**0**	**0**	**0**	**0**	**0**	**0**	**0**	**0**	**1**	**0**

**Abbreviations:** N/A = information not applicable because treatment is not covered; No = barrier does not apply to any Medicaid enrollee; V = varies, with barrier applying to some, but not all, Medicaid enrollees; Yes = barrier applies to all Medicaid enrollees.

* Data as of December 31, 2008, and December 31, 2018.

^†^ Because of differences in the methods and timing of data collection, some findings differ from findings on this topic published before 2014.

^§^ Barriers apply to one or more cessation treatments.

The number of states having none of the seven barriers to cessation treatment increased from zero to two during December 31, 2008–December 31, 2018. During this period, the number of states that did not require copayments for any cessation treatment for any traditional Medicaid enrollees approximately tripled, from 10 to 28. As of December 31, 2018, states reported that the most common barriers imposed on all or some traditional Medicaid enrollees were limits on duration of treatment (44 states, 86%), annual limits on quit attempts (37, 72%), and prior authorization requirements (35, 69%) (Table 3).

## Discussion

States made substantial progress in improving Medicaid coverage of proven tobacco cessation treatments during 2008–2018, with the number of states covering all nine cessation treatments for all traditional Medicaid enrollees increasing from six to 15 and the number of states covering all seven FDA-approved cessation medications increasing from 20 to 36. Improved coverage increases Medicaid enrollees’ access to cessation treatments, which can make it easier for them to quit smoking (*3*,*5*,*6*). Covering all nine cessation treatments is important because different smokers respond better to or prefer different treatments than do other smokers.

The increase in the number of states covering all nine cessation treatments likely resulted in part from the Patient Protection and Affordable Care Act (ACA), which was passed in March 2010 (*3*). Two provisions of the ACA that introduced new requirements for state Medicaid cessation coverage took effect during the study period. The first provision, which took effect in October 2010, requires state Medicaid programs to cover cessation counseling and FDA-approved cessation medications for pregnant women with no cost-sharing^¶¶^; this provision resulted in increases in state Medicaid coverage of cessation counseling and medications for pregnant women (*8*). The second provision, which took effect in January 2014, barred state Medicaid programs that participate in the Medicaid drug rebate program from excluding FDA-approved cessation medications from coverage.*** This provision likely contributed to the increase observed in this study in the number of states that cover all seven FDA-approved cessation medications (*3*). With the exception of pregnant Medicaid enrollees, the ACA does not require traditional state Medicaid programs to cover cessation counseling or to remove barriers that impede access to cessation counseling and medications.^†††^

Although the progress reported here is encouraging, state Medicaid cessation coverage still falls short of the Healthy People 2020 objective of comprehensive cessation coverage in all 50 states and DC.^§§§^ In particular, state Medicaid coverage of counseling lags behind medication coverage, with the number of states that cover both individual and group counseling only increasing from 13 states in 2008 to 16 states in 2018. The combined use of counseling and medication is more effective in increasing quit rates than is the use of either of these treatments alone (*3*). Combined state Medicaid coverage of cessation counseling and medications has been found to be associated with an estimated mean increase in past-year quitting of 3.0 percentage points compared with that in persons without such coverage (*5*).

In addition, as of December 2018, all but two states retained barriers that make it more difficult for Medicaid enrollees to access cessation treatments. Removing these barriers would further increase Medicaid enrollees’ access to and use of cessation treatments (*3*,*6*). Although state Medicaid programs made considerable progress in removing copayments during the study period, progress in removing other barriers was mixed.

State Medicaid cessation coverage often varies considerably across a state’s Medicaid managed care plans in terms of both cessation treatments covered and coverage barriers. Standardizing cessation coverage by having all managed care plans cover all proven cessation treatments with minimal barriers can be beneficial in maximizing Medicaid enrollees’ access to proven cessation treatments while minimizing confusion about coverage among enrollees and providers. Standardizing coverage in this way is especially important because states are increasingly moving Medicaid enrollees from fee-for-service coverage into managed care coverage.^¶¶¶^

The findings in this report are subject to at least two limitations. First, when official documents were not publicly available or were outdated or conflicting, state government personnel were contacted for clarification; however, it was not always possible to verify the accuracy of the information they provided. Second, cessation coverage can vary widely across Medicaid managed care plans and can change with little notice, which makes determining these plans’ coverage challenging.

Approximately 6.7 million adult smokers report being enrolled in Medicaid, accounting for approximately 20% of adult U.S. cigarette smokers.**** Whereas smokers enrolled in Medicaid are as likely as are privately insured smokers to want to quit and to make a past-year quit attempt, they are less likely to succeed in quitting (*9*,*10*). Compared with smokers with private health insurance, smokers enrolled in Medicaid have been found to be more likely to have chronic diseases and to experience severe psychological distress (*9*). The high smoking prevalence among Medicaid enrollees imposes a substantial health burden on these persons and on society, and is a major driver of federal and state health care expenditures (*3*). Smoking-related diseases accounted for approximately 15% of annual Medicaid spending during 2006–2010, amounting to approximately $39 billion in 2010 (*2*). State Medicaid programs can help reduce this health and financial burden by covering all evidence-based cessation treatments, removing coverage barriers, and promoting covered treatments to Medicaid enrollees and providers to increase their use (*3*–*7*).

**Summary**

**What is already known about this topic?**

Medicaid enrollees have a higher prevalence of cigarette smoking than do privately insured U.S. residents. Evidence indicates that comprehensive, barrier-free state Medicaid cessation coverage could reduce smoking, smoking-related disease, and health care expenditures among Medicaid enrollees.

**What is added by this report?**

As of December 31, 2018, 15 states covered all nine evidence-based cessation treatments for all traditional Medicaid enrollees, up from six states at the end of 2008. All but two states retained coverage barriers.

**What are the implications for public health practice?**

State Medicaid programs can help Medicaid enrollees quit smoking by covering all evidence-based cessation treatments, removing coverage barriers, and promoting treatments to increase their use.

## Footnotes

* FDA has approved seven medications for smoking cessation, including five nicotine replacement therapies (the nicotine patch, gum, lozenge, nasal spray, and inhaler) and two nonnicotine medications (bupropion and varenicline).

† As used in this report, the term “traditional” Medicaid enrollees refers to persons who are enrolled in Medicaid under traditional Medicaid eligibility criteria (e.g., low-income pregnant women, children and persons with a disability), as opposed to the income-only eligibility criteria (i.e. income equal or less than 138% of the federal poverty level) for coverage under expanded Medicaid, created by the Patient Protection and Affordable Care Act and implemented in 2014. https://www.healthcare.gov/medicaid-chip/getting-medicaid-chip/.

§ Telephone counseling is available free to callers to state quitlines (including Medicaid enrollees) in all 50 states and DC through the national quitline portal 1-800-QUIT-NOW and was not included in this report. In June 2011, the Centers for Medicare & Medicaid Services announced that it would offer a 50% federal administrative match to state Medicaid programs for the cost of state quitline counseling provided to Medicaid enrollees.

¶ These seven coverage barriers are requirement of copayment, requirement of prior authorization, requirement of counseling for medications, stepped care therapy, limits on duration, annual limit on number of covered quit attempts, and lifetime limit on number of covered quit attempts. States were considered to have a barrier if that barrier was in place for one or more cessation treatments.

** Information on state Medicaid cessation coverage compiled by the American Lung Association is available in the CDC State Activities Tracking and Evaluation (STATE) System, a database that contains tobacco-related epidemiologic and economic data and information on state tobacco-related legislation (https://www.cdc.gov/statesystem). Certain data presented in this report differ slightly from Medicaid cessation coverage data reported in the STATE System because of small differences in coding rules, categories, and reporting periods.

†† As used in this report, the term “states” includes DC.

§§ These states are no longer considered to provide comprehensive Medicaid cessation coverage because of changes in coverage in their Medicaid managed care plans.

¶¶ Patient Protection and Affordable Care Act, Pub. L. 111–48 124 Stat. 560, March 23, 2010, as amended through May 1, 2010 (https://www.congress.gov/111/plaws/publ148/PLAW-111publ148.pdf). The Centers for Medicare & Medicaid Services has issued guidance to states on implementing this provision (https://downloads.cms.gov/cmsgov/archived-downloads/SMDL/downloads/SMD11-007.pdf).

*** Patient Protection and Affordable Care Act, Pub. L. 114–48 124 Stat. 310, March 23, 2010, as amended through May 1, 2010 (https://www.congress.gov/111/plaws/publ148/PLAW-111publ148.pdf). The Centers for Medicare & Medicaid Services has issued guidance to states on implementing this provision (https://www.medicaid.gov/Medicaid-CHIP-Program-Information/By-Topics/Prescription-Drugs/Downloads/Rx-Releases/State-Releases/state-rel-165.pdf). As of December 31, 2019, the Centers for Medicare & Medicaid Services had published state plan amendments from 38 states declaring that they had implemented this provision. As of December 31, 2018, all 50 states and DC participated in the Drug Rebate program.

††† Unlike traditional Medicaid coverage, expanded Medicaid is required to include coverage without cost-sharing of preventive services receiving an A or B rating from the U.S. Preventive Services Task Force (USPSTF). Tobacco cessation intervention has received an A rating from USPSTF. https://www.ncbi.nlm.nih.gov/pmc/articles/PMC4265933/.

§§§ https://www.healthypeople.gov/2020/topics-objectives/topic/tobacco-use/objectives.

¶¶¶ https://www.kff.org/medicaid/state-indicator/share-of-medicaid-population-covered-under-different-delivery-systems/?currentTimeframe.

**** This estimate includes enrollees in both traditional and expanded Medicaid, as determined in the 2018 National Health Interview Survey conducted by CDC’s National Center for Health Statistics. https://www.cdc.gov/nchs/nhis/data-questionnaires-documentation.htm.
